# Reduced interocular suppression after inverse patching in anisometropic amblyopia

**DOI:** 10.3389/fnins.2023.1280436

**Published:** 2023-12-11

**Authors:** Jingyi Hu, Jing Chen, Yixuan Ku, Minbin Yu

**Affiliations:** ^1^State Key Laboratory of Ophthalmology, Guangdong Provincial Key Laboratory of Ophthalmology and Visual Science, Zhongshan Ophthalmic Center, Sun Yat-sen University, Guangzhou, China; ^2^School of Psychology, Shanghai University of Sport, Shanghai, China; ^3^Center for Brain and Mental Wellbeing, Department of Psychology, Sun Yat-sen University, Guangzhou, China; ^4^Peng Cheng Laboratory, Shenzhen, China

**Keywords:** suppression, inverse patching, anisometropic amblyopia, amblyopia, steady-state visual evoked potentials

## Abstract

**Purpose:**

Recent investigations observed substantial enhancements in binocular balance, visual acuity, and stereovision among older children and adults with amblyopia by patching the amblyopic eye (i.e., inverse patching) for 2 h daily over 2 months. Despite these promising findings, the precise neural mechanisms underlying inverse patching remain elusive. This study endeavors to delve deeper into the neural alterations induced by inverse patching, focusing on steady-state visual evoked potentials (SSVEPs). We specifically investigate the changes in SSVEPs following monocular deprivation of either the fellow eye or the amblyopic eye in older amblyopic children and adults.

**Method:**

Ten participants (17.60 ± 2.03 years old; mean ± SEM), clinically diagnosed with anisometropic amblyopia, were recruited for this study. Each participant underwent a 120 min patching session on their fellow eye on the first day, followed by a similar session on their amblyopic eye on the second day. Baseline steady-state visual evoked potentials (SSVEPs) measurements were collected each day prior to patching, with post-patching SSVEPs measurements obtained immediately after the patching session. The experimental design incorporated a binocular rivalry paradigm, utilizing SSVEPs measurements.

**Results:**

The results revealed that inverse patching induced a heightened influence on neural plasticity, manifesting in a reduction of interocular suppression from the fellow eye to the amblyopic eye. In contrast, patching the fellow eye demonstrated negligible effects on the visual cortex. Furthermore, alterations in interocular suppression subsequent to inverse patching exhibited a correlation with the visual acuity of the amblyopic eye.

**Conclusion:**

Inverse patching emerges as a promising therapeutic avenue for adolescents and adults grappling with severe anisometropic amblyopia that proves refractory to conventional interventions. This innovative approach exhibits the potential to induce more robust neural plasticity within the visual cortex, thereby modulating neural interactions more effectively than traditional amblyopia treatments.

## Introduction

Amblyopia represents a pathological condition characterized by compromised visual information processing (Hubel and Wiesel, 1970; Holmes and Clarke, 2006), frequently accompanied by diminished visual acuity and various forms of visual dysfunction (McKee et al., 2003; Kelly et al., 2019; Hu et al., 2021). Extensive research has delved into the visual impairment associated with amblyopia, revealing aberrant binocular connections in the visual cortex of individuals with amblyopia (Wiesel and Hubel, 1963; Goodyear et al., 2000; Barnes et al., 2001; Schmidt et al., 2004; Baker et al., 2015; Acar et al., 2019).

Historically, amblyopia has been addressed through interventions such as patching, atropine administration, and filters to compel the amblyopic eye to engage in visual processing, concurrently diminishing visual input in the fellow eye (Pediatric Eye Disease Investigator Group, 2002; Loudon and Simonsz, 2005). Currently, the primary clinical approach for unilateral amblyopia involves patching the fellow eye (Repka et al., 2003). This method reduces sensory inputs and neural activity in the visual cortex from the fellow eye, demonstrably enhancing visual acuity in the amblyopic eye (Hubel and Wiesel, 1968; Tigges et al., 1992). However, the efficacy of patching treatment for amblyopia diminishes in older patients compared to their younger counterparts (Scheiman et al., 2005; Holmes et al., 2011). While it is widely acknowledged that neural plasticity declines with age in children (Hensch, 2005; Hensch and Quinlan, 2018; Abuleil et al., 2019), the impact of patching on neural interactions in the visual cortex of older patients remains unclear.

Recent investigations have explored alternative strategies to enhance monocular and binocular visual functions in older amblyopic children and adults. These approaches include perceptual learning, inverse patching, and noninvasive brain stimulation (Li et al., 2013; Chen et al., 2016; Ding et al., 2016; Liu and Zhang, 2018; Zhou et al., 2019). Decades ago, early studies indicated that inverse patching, involving the patching of the amblyopic eye, was considered less effective compared to conventional occlusion, resulting in its discontinuation for an extended period (Noordeng, 1965; Malik et al., 1970). However, recent investigations by Lunghi et al. (2018) and Zhou et al. (2019) have demonstrated that inverse occlusion significantly enhances binocular balance, visual acuity, and stereovision in older amblyopic children and adults. This outcome suggests a potential for substantial neural plasticity within the visual cortex of older amblyopic individuals through inverse patching.

To elucidate the neural underpinnings of such plasticity, it is crucial to explore the efficacy of inverse patching using electrophysiological techniques. The application of steady-state visual evoked potentials (SSVEPs), primarily originating from the primary visual cortex, provides a reliable and efficient method to investigate cortices with a high signal-to-noise ratio (Chen et al., 2015; Norcia et al., 2015; Min et al., 2021). Examination of SSVEP patterns of neural activity in the visual cortices of amblyopic adults has revealed abnormalities in activation and impaired binocular visual functions (Baker et al., 2015; Chadnova et al., 2017; Lygo et al., 2021). Additionally, SSVEPs have proven instrumental in elucidating heightened responses during short-term monocular patching (Zhou et al., 2015) and the impacts of monocular perceptual learning (Gu et al., 2020).

The present study aims to assess the neural efficacy of short-term inverse patching in older children (age >11 years old, Epelbaum et al., 1993; Fronius et al., 2014) and younger adults with amblyopia. We employ SSVEPs to quantify alterations in neural responses following monocular deprivation of either the fellow eye or the amblyopic eye. Furthermore, we intend to examine potential correlations between neural indexes and visual acuity in the context of older amblyopic children and adults.

## Methods

### Participants

Ten anisometropic amblyopic participants were recruited from the Zhongshan Ophthalmic Center (5 females and 5 males; ages 12 to 29 years; mean ± SEM, 17.60 ± 2.03 years). Clinical data for these participants are presented in Table 1. The best-corrected visual acuity (BCVA) of the fellow eye was significantly superior to that of the amblyopic eye (BCVA of the fellow eye: 0.00 ± 0.00 logMAR; BCVA of the amblyopic eye: 0.48 ± 0.09 logMAR; Wilcoxon signed-rank test: *z* = −3.19, *p* < 0.01).

**Table 1:** Clinical information on patients with anisometropic amblyopia.

Subjects	Age	Sex	BCVA of fellow eye (logMAR)	BCVA of amblyopic eye (logMAR)	Interocular refractive difference (diopter)	Stereoacuity (arcsec)
1	12	M	0	0.2	2.125	N
2	13	F	0	0.2	4.25	N
3	21	F	0	0.2	4.5	200
4	12	F	0	0.3	4.5	500
5	17	M	0	0.3	2	200
6	28	M	0	0.4	5	100
7	16	F	0	0.7	5.375	N
8	29	M	0	0.7	2.25	N
9	16	M	0	0.8	2.625	N
10	12	F	0	1	2.5	N

N, non measurable.

This study received ethical approval from the Zhongshan Ophthalmic Center Ethics Committee, adhering to the principles outlined in the Declaration of Helsinki. Prior to data collection, informed consent forms were duly signed by all participants or their legal guardians. Inclusion criteria for anisometropic amblyopia participants comprised the following: (1) a best-corrected visual acuity (BCVA) difference between eyes equal to or exceeding 2 lines; (2) an interocular refractive difference of spherical equivalent or astigmatism of 1.0 diopter (D) or more; (3) a documented history of anisometropic amblyopia without concurrent disorders; (4) normal color vision.

Before the commencement of the study, all participants underwent a comprehensive ophthalmologic examination and received refractive correction based on their cycloplegic refraction. The ophthalmologic examination encompassed cycloplegic refraction, slit-lamp examination, funduscopic examination, best-corrected distance visual acuity (BCVA) measured using the Early Treatment Diabetic Retinopathy Study numbers chart, and stereoacuity Randot Stereotests (Stereo Optical Company, Inc., Chicago, IL, United States). BCVA measurements were transformed to log MAR.

### Experimental procedure

Participants were instructed to discontinue atropine or patching treatment at least 1 week before the commencement of the experiment. Prior to steady-state visual evoked potentials (SSVEPs) measurements, assessments of best-corrected visual acuity (BCVA) and stereopsis were conducted. On the first experimental day, each participant underwent baseline SSVEPs measurements. Subsequently, the participant was instructed to patch the fellow eye with a translucent patch, eliminating contour information and reducing transmittance by 20%, for a duration of 120 min. Post-patching SSVEPs measurements were conducted immediately following the removal of the patch from the fellow eye.

**Figure 1:**
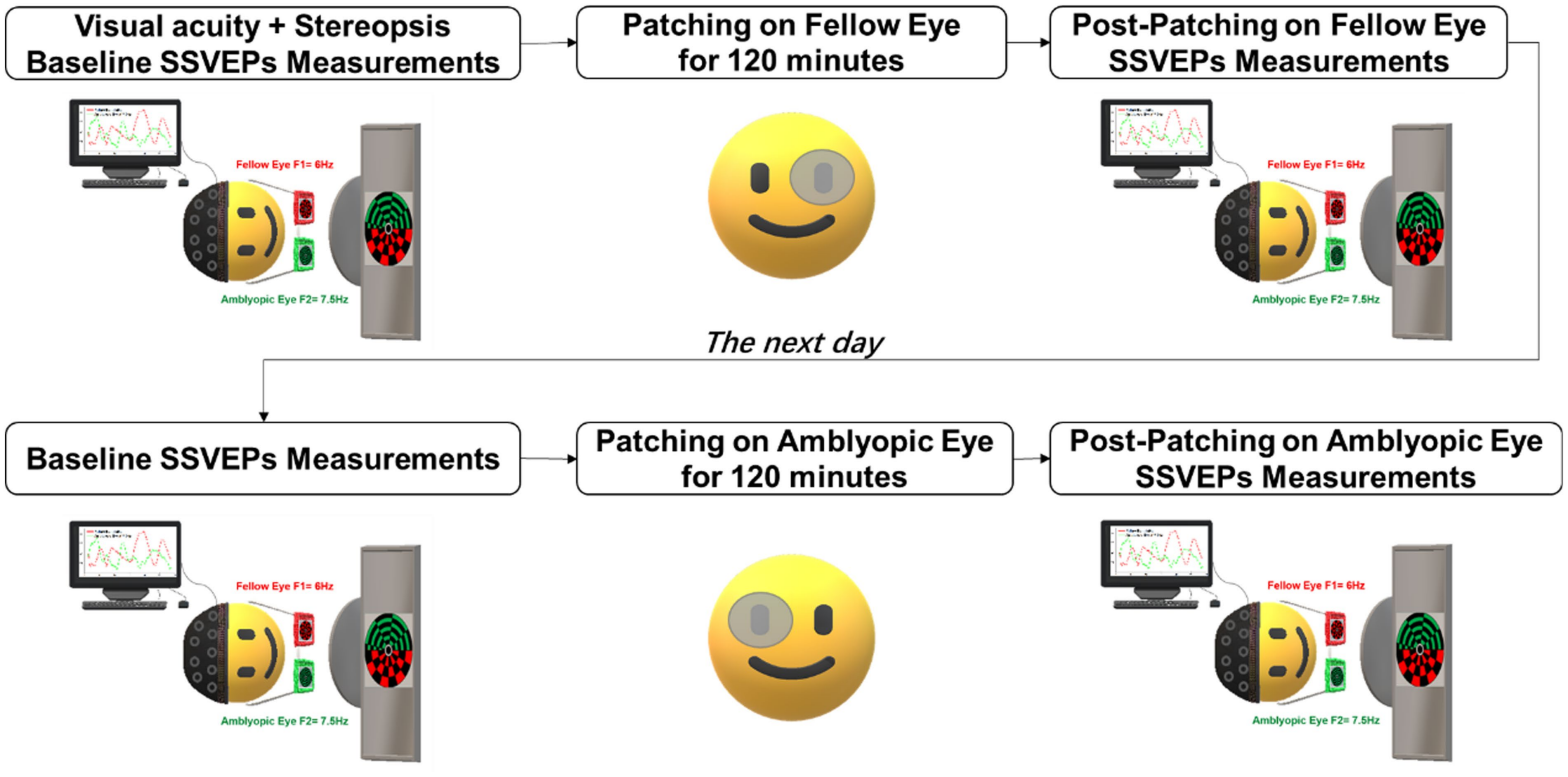
Experimental procedure. Participants underwent short-term patching on the fellow eye for 2 h on 1 day and inverse patching (patching of the amblyopic eye) for 2 h on the subsequent day. SSVEPs measurements were recorded both before and after each patching session while the subjects viewed flickering binocular rivalry stimuli.

On the second day, participants returned for the inverse patching session, involving patching of the amblyopic eye. Similar to the first day, participants underwent a baseline SSVEPs test, followed by 120 min of inverse patching. Post-patching SSVEPs measurements were performed (refer to Figure 1 for a visual representation of the experimental timeline). All participants actively engaged in all four sessions of SSVEPs measurements throughout the experiment. Each SSVEPs measurement session comprised 18 trials, with each trial presenting 30 s of binocular rivalry stimuli.

### Binocular rivalry stimuli

**Figure 2:**
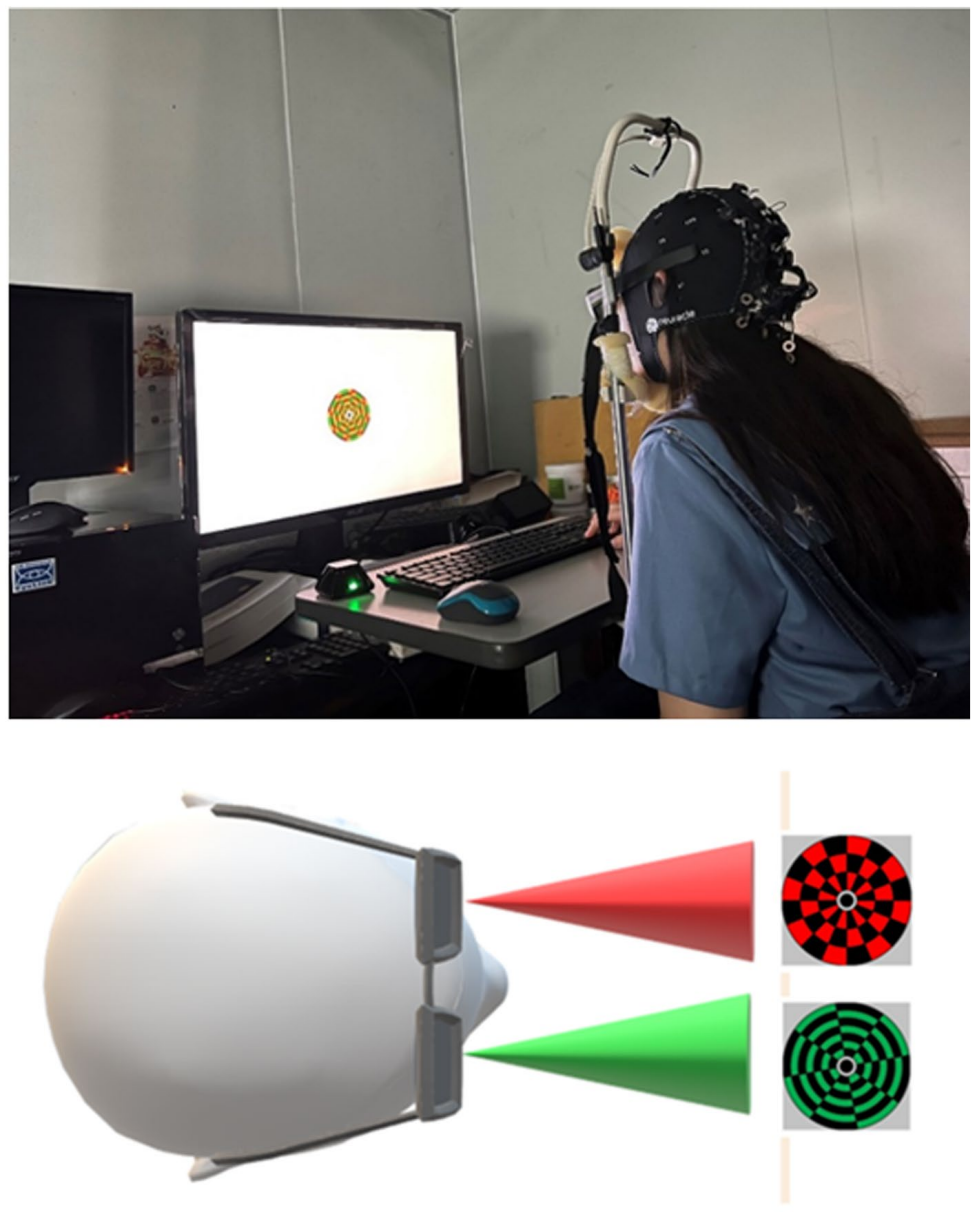
Binocular rivalry stimuli. Binocular stimuli, differentiated by color, were simultaneously presented to each eye.

Binocular rivalry stimuli were presented to participants using stereo goggles (NVIDIA 3D Vision 2) on a gamma-corrected 27-inch ASUS VG278HE monitor, with a mean luminance of 150 cd/m^2^, situated in a dark and shielded environment. Throughout the experiment, a chinrest positioned participants at 57 cm from the screen, minimizing head movements. A pair of circular checkerboards, each within a 10° visual angle, were concurrently presented to the fellow eye at 6 Hz and the amblyopic eye at 7.5 Hz. These stimuli were generated and displayed using MATLAB (Mathworks, Natick, MA, United States) and Psychtoolbox (Pelli, 1997). Participants’ task during the experiment was to focus on the central region of the circular checkerboards presented to each eye throughout the trials. To ensure experimental control and precise alignment, participants were directed to concentrate on the central region of the stimuli. To maintain precise alignment, all participants in this study calibrated both eyes’ stimuli before SSVEPs measurements. This alignment was achieved by viewing a single circular checkerboard in which red and green colors alternated (refer to Figure 2).

### EEG acquisition and data analysis

Our study recorded SSVEP signals at occipital areas, specifically at electrodes Oz, O1, O2, Pz, P3, P4, PO3, and PO4, following the guidelines by Norcia et al. (2015). A 32-channel wireless EEG system (NeuSen.W32, Neuracle, China) was employed for data acquisition, with a sampling rate set at 1000 Hz. The ground electrode was positioned on AFz, and the reference electrode on CPz, with the impedance of each electrode meticulously maintained below 10 kΩ.

The SSVEP data underwent analysis using MATLAB and EEGLAB (Delorme and Makeig, 2004). To minimize noise, a Laplacian spatial filter was applied to the raw data by subtracting the signal at Oz from the average of the signals at adjacent electrodes (Pz, P3, P4, PO3, PO4, O1, O2). The collected EEG signals were subsequently segmented into 30 s epochs and band-pass filtered using a finite impulse response (FIR) filter within the frequency range of 1 to 30 Hz. SSVEP amplitudes at specific frequencies (f1 = 6 Hz for the fellow-eye frequency, f2 = 7.5 Hz for the amblyopic-eye frequency, and f1 + f2 = 13.5 Hz for the intermodulation response) were measured employing the recursive least-squares adaptive filter (RLS) technique (Tang and Norcia, 1995).

Technical issues led to the exclusion of a minimal proportion of trials (2.6% of all trials), such as those with missing recording markers. Consequently, 15 to 18 epochs were collected for each participant for subsequent analysis. The two fundamental frequencies (f1 = 6 Hz for the fellow eye and f2 = 7.5 Hz for the amblyopic eye) that exhibited significant responses at the corresponding frequencies (both signal-to-noise ratios >1, *p* < 0.01) were employed to identify the neural response to each eye. The intermodulation frequency (IM, f1 + f2 = 13.5 Hz) represented visual activities related to interocular interaction, surpassing noise levels significantly (signal-to-noise ratio >1, *p* < 0.01). Normalization of neural amplitudes for each participant was performed concerning the amplitude of the fellow-eye frequency and the amblyopic-eye frequency to mitigate inter-subject variability


$$\begin{array}{l} \mathrm{Interocular}\;\mathrm{suppression}\\ \;=\left({\mathrm{Amplitude}}_{\mathrm{Fellow}\;\mathrm{eye}}-{\mathrm{Amplitude}}_{\mathrm{Amblyopic}\;\mathrm{eye}}\right)\\ \;/\left({\mathrm{Amplitude}}_{\mathrm{Fellow}\;\mathrm{eye}}+{\mathrm{Amplitude}}_{\mathrm{Amblyopic}\;\mathrm{eye}}\right) \end{array}$$

### Statistical analysis

Statistical analyses were conducted using SPSS Version 22, with a predetermined level of statistical significance set at *p* < 0.05 for all analyses. The Wilcoxon signed-rank test was employed to compare visual acuity, stereoacuity, and patching effects observed in the SSVEPs data. Additionally, the Spearman rank correlation was utilized to explore relationships between variables. All presented data are expressed as mean ± SEM.

## Results

### SSVEP responses at baseline in amblyopia

**Figure 3:**
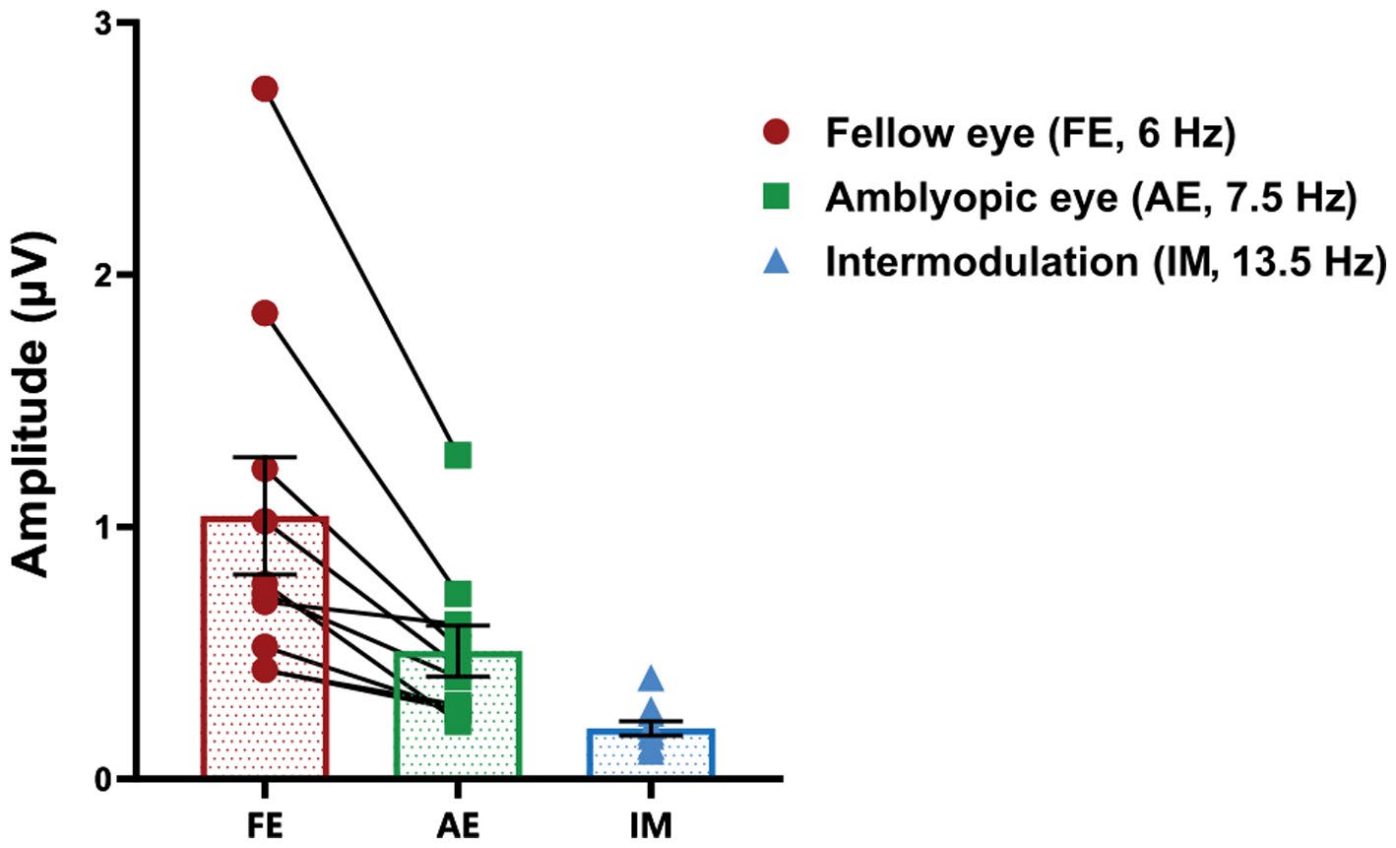
SSVEP responses at baseline in anisometropic amblyopes. The mean amplitude of SSVEP responses induced by the fellow-eye frequency (red, f1 = 6 Hz), amblyopic-eye frequency (green, f2 = 7.5 Hz), and intermodulation frequency (blue, f1 + f2 = 13.5 Hz) is presented. Data are expressed as mean ± SEM.

We computed the SSVEP responses during baseline measurements prior to patching. The mean amplitude of SSVEP responses induced by the fellow-eye frequency (f1 = 6 Hz), amblyopic-eye frequency (f2 = 7.5 Hz), and intermodulation frequency were illustrated in Figure 3. Additionally, there were no significant differences in baseline SSVEP responses and interocular suppression when comparing the first day to the second day in our study (*p* > 0.05).

### Short-term inverse patching affects neural responses in amblyopia

**Figure 4:**
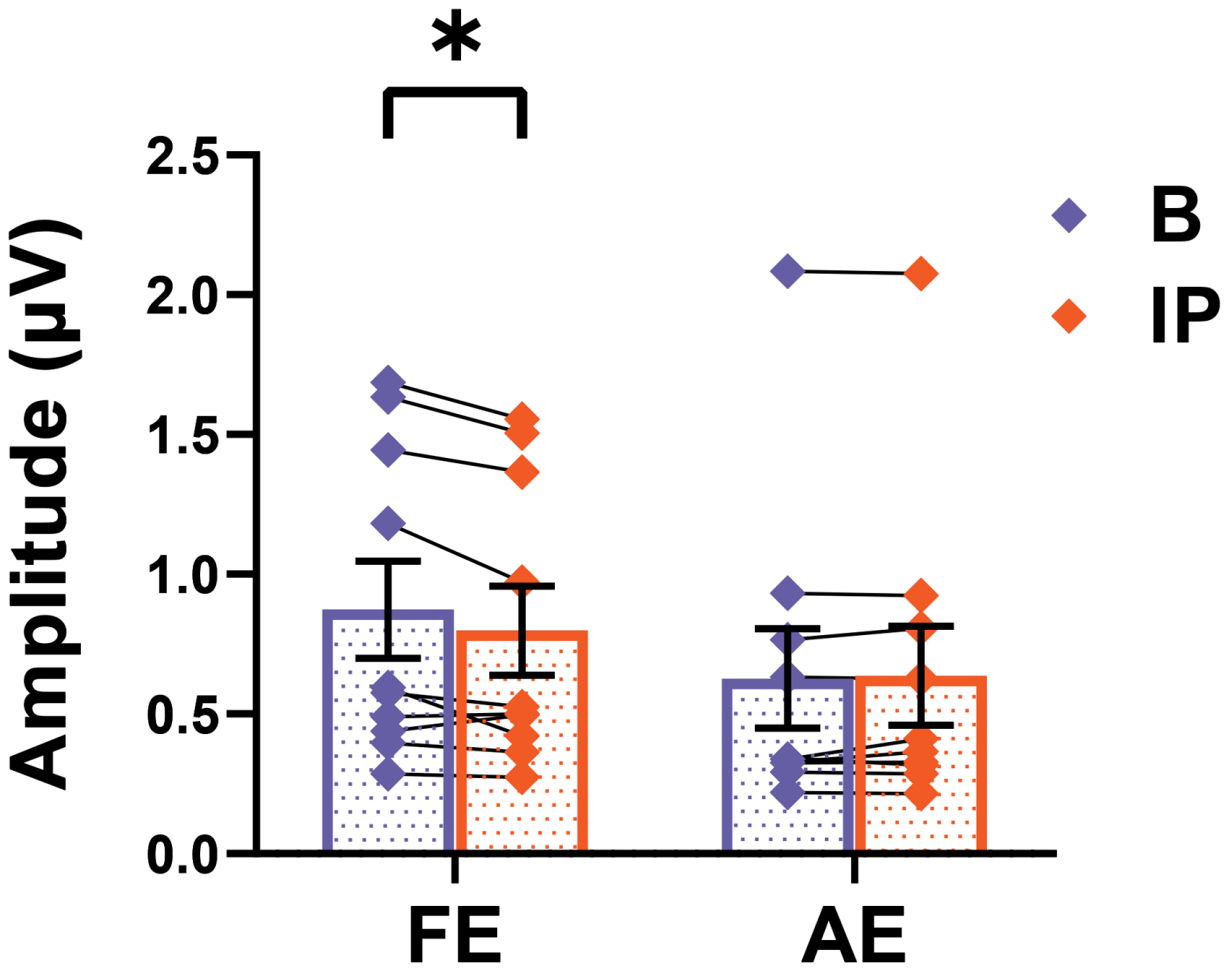
SSVEP responses alters after short-term inverse patching. The SSVEP responses from the fellow eye and the amblyopic eye were measured both during baseline (B) and after short-term inverse patching (IP). Data are presented as mean ± SEM, with asterisks denoting statistical significance. ^*^p < 0.05.

We assessed SSVEP responses during baseline and post-patching periods in all amblyopic participants to investigate the impact of short-term monocular deprivation on the amblyopic eye. As depicted in Figure 4, the amplitude of SSVEP response induced by the frequency of visual stimulation in the fellow eye significantly decreased after short-term inverse patching (fellow eye: baseline 0.87 ± 0.17 μV; after short-term inverse patching 0.80 ± 0.16 μV; Wilcoxon signed-rank test: *z* = −2.19, *p* < 0.05). Conversely, the amplitude of SSVEP response induced by the visual stimulation frequency of the amblyopic eye did not change significantly (amblyopic eye: baseline 0.63 ± 0.18 μV; after short-term inverse patching 0.64 ± 0.18 μV; Wilcoxon signed-rank test: *z* = −0.51, *p* = 0.96). No significant difference was observed in the amplitude of SSVEP response induced by intermodulation frequency before and after short-term inverse patching (intermodulation: baseline 0.21 ± 0.03 μV; after short-term inverse patching 0.22 ± 0.03 μV; Wilcoxon signed-rank test: *z* = −1.78, *p* = 0.07).

**Figure 5:**
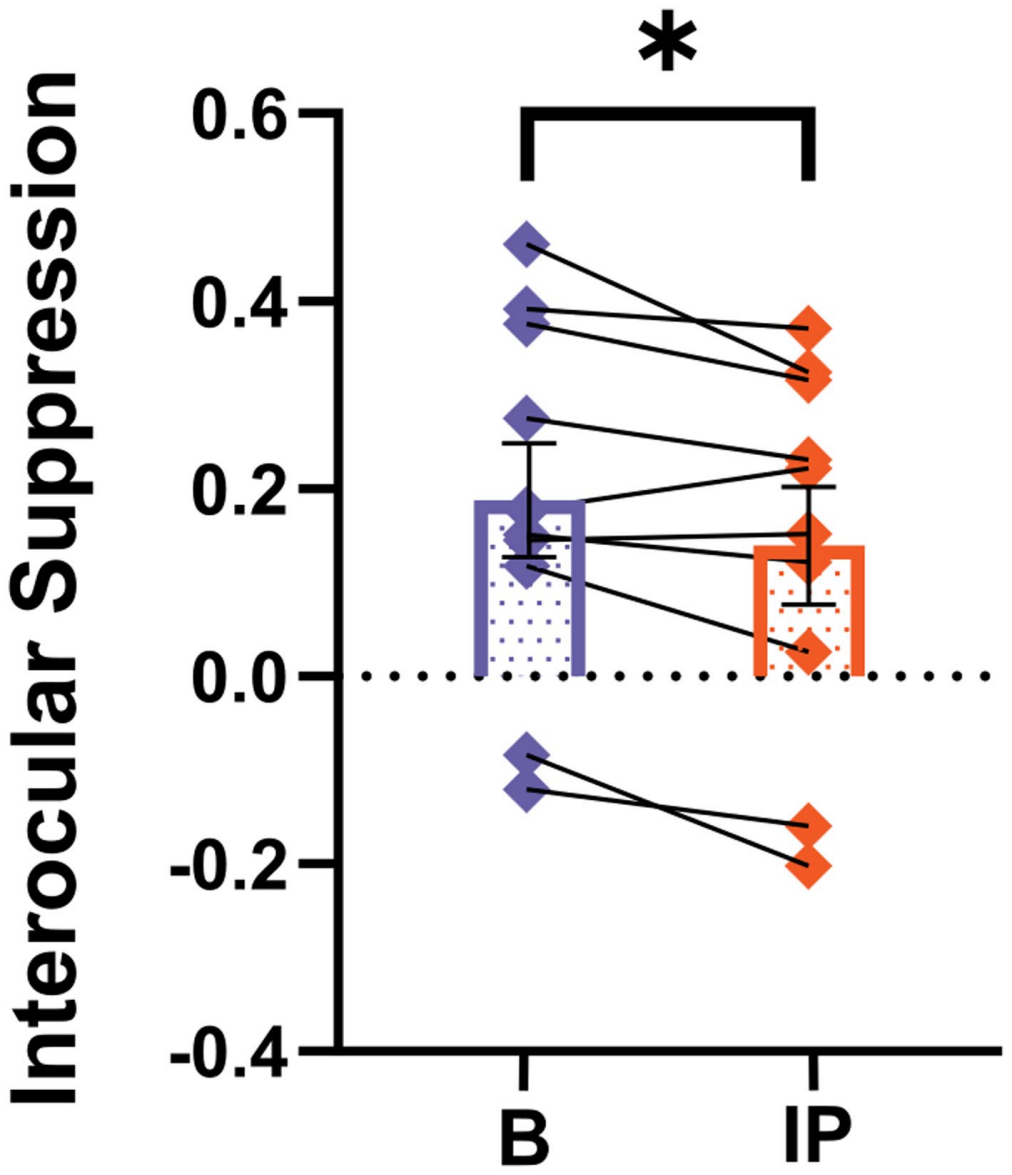
SSVEP neural index differs after short-term inverse patching. Interocular suppression was quantified both during baseline (B) and after short-term inverse patching (IP) in amblyopes. Data are presented as mean ± SEM, with asterisks indicating statistical significance. ^*^p < 0.05.

We proceeded to compare interocular suppression in the visual cortex before and after inverse patching, as illustrated in Figure 5. The interocular suppression from the fellow eye toward the amblyopic eye significantly decreased after the amblyopic eye was patched for 2 h (baseline: 0.19 ± 0.06; after short-term inverse patching: 0.14 ± 0.06; Wilcoxon signed-rank test: *z* = −2.09, *p* < 0.05).

**Figure 6:**
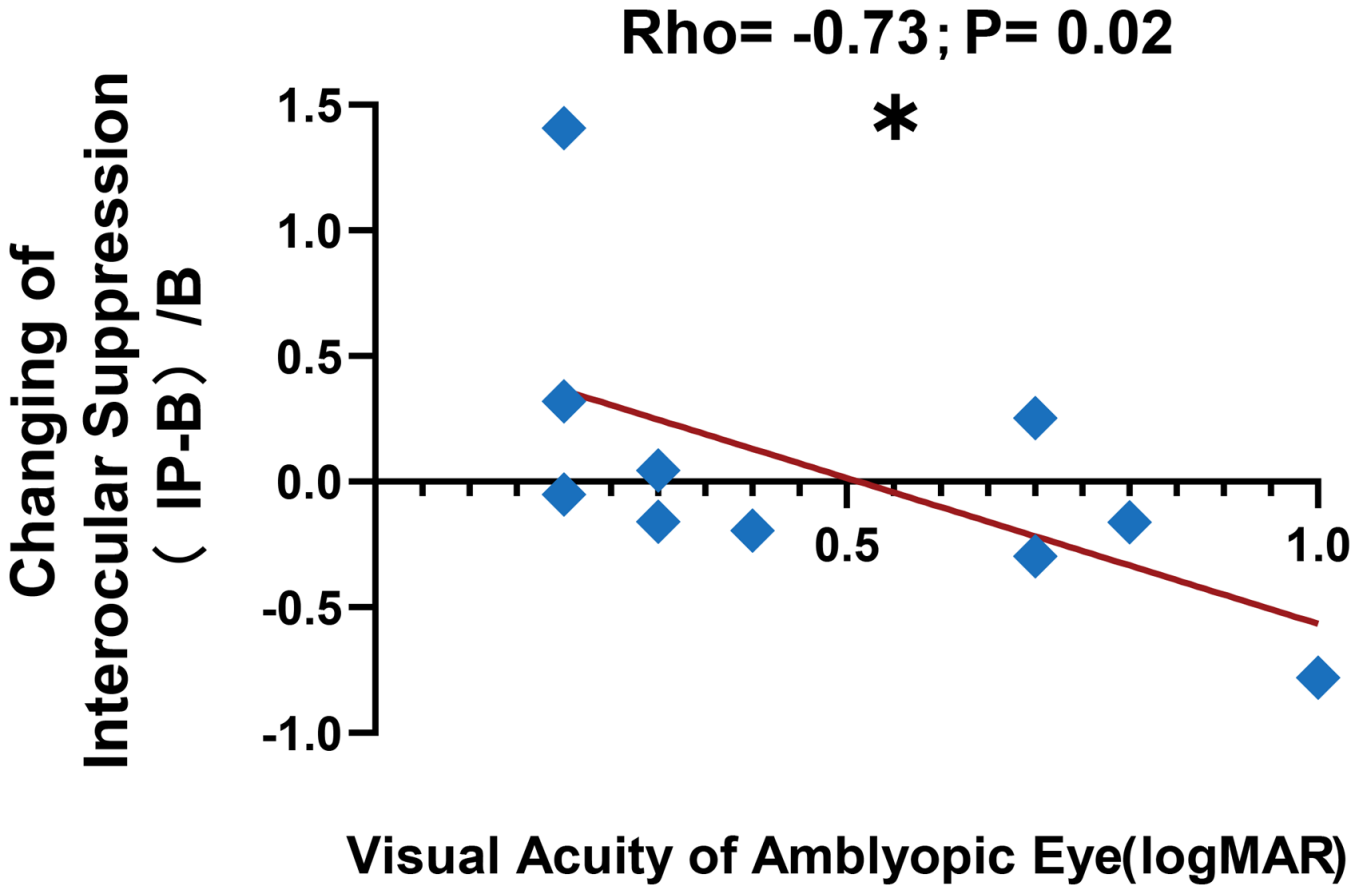
Correlation after short-term inverse patching. We observed a negative correlation between the visual acuity of the amblyopic eye in logMAR and the change in interocular suppression after short-term inverse patching in amblyopes, as illustrated by the blue diamond symbols and the red line in this figure. The formula used for the calculation is denoted as (IP − B)/B, where (Interocular Suppression after Inverse Patching − Interocular Suppression in Baseline)/Interocular Suppression in Baseline. Data are presented as mean ± SEM, with asterisks indicating statistical significance. ^*^p < 0.05.

Moreover, we observed a correlation between the visual acuity of the amblyopic eye and the change in interocular suppression after patching the amblyopic eye, as depicted in Figure 6 (change in interocular suppression: 0.04 ± 0.18; visual acuity of amblyopic eye: 0.48 ± 0.09 logMAR; Spearman rank correlation: Rho = −0.73, *p* < 0.05). These findings suggest that anisometropic amblyopia with worse visual acuity in the amblyopic eye manifests a more pronounced alteration in interocular suppression after short-term monocular deprivation in the amblyopic eye, gravitating more toward binocular equilibrium following inverse patching.

### Short-term patching of fellow eye in amblyopia

We conducted a thorough evaluation of SSVEP responses between baseline and post-patching periods in all amblyopic participants to examine the impact of short-term monocular deprivation on the fellow eye. Our analysis revealed no significant differences before and after short-term patching on the fellow eye in SSVEP amplitudes induced by the frequency of visual stimulation in the fellow eye (fellow eye: baseline 1.04 ± 0.23 μV; after short-term patching on the fellow eye 0.99 ± 0.20 μV; Wilcoxon signed-rank test: *z* = −0.15, *p* = 0.88), amblyopic eye (amblyopic eye: baseline 0.51 ± 0.10 μV; after short-term patching on the fellow eye 0.47 ± 0.08 μV; Wilcoxon signed-rank test: *z* = −0.56, *p* = 0.58), and intermodulation frequency (intermodulation: baseline 0.20 ± 0.03 μV; after short-term patching on the fellow eye 0.20 ± 0.03 μV; Wilcoxon signed-rank test: *z* = −0.46, *p* = 0.65). Furthermore, there were no significant changes in interocular suppression (interocular suppression: baseline 0.32 ± 0.04; after short-term patching on the fellow eye 0.34 ± 0.04; Wilcoxon signed-rank test: *z* = −0.66, *p* = 0.51) after short-term patching on the fellow eye. These results indicate notable differences in SSVEP response amplitude and neural indices between short-term monocular deprivation on the fellow eye and on the amblyopic eye in older children and adults with anisometropic amblyopia.

## Discussion

In this study, we conducted an electrophysiological investigation utilizing steady-state visual evoked potentials (SSVEPs) to elucidate the impact of inverse patching on neural responses in older anisometropic amblyopic children and younger adults. Our results revealed discernible differences in neural plasticity and responses to patching on different eyes in anisometropic amblyopes. Specifically, inverse patching exerted a more substantial effect on neural response and indices, whereas patching the fellow eye demonstrated no significant impact on neural activity. Additionally, we identified a correlation between neural changes in interocular interactions after various short-term patching methods and the visual acuity of the amblyopic eye in anisometropic amblyopia.

These findings underscore the necessity for a nuanced approach in selecting amblyopic treatment modalities, taking into account factors such as age and tailoring interventions based on individual neural responses. Notably, inverse patching emerged as a promising therapeutic option for older adolescents and adults with amblyopia, particularly in cases where traditional patching treatments prove ineffective.

Prior research has suggested that short-term monocular deprivation can induce alterations in ocular dominance plasticity (Lunghi et al., 2011; Zhou et al., 2013; Lunghi et al., 2015a,b; Chadnova et al., 2017; Spiegel et al., 2017). Our study extends these findings by demonstrating that adolescent and adult amblyopic patients exhibit preserved neuroplasticity when subjected to inverse patching, contrasting with the less favorable outcomes observed with traditional patching. The diminished plasticity of ocular dominance induced by short-term patching on the fellow eye in adolescent and adult amblyopes aligns with clinical observations that older patients derive lesser benefits from traditional patching treatments compared to their younger counterparts (Rutstein and Fuhr, 1992; Epelbaum et al., 1993; Holmes et al., 2011; Fronius et al., 2014). However, it is crucial to note that traditional patching of the fellow eye retains therapeutic value for amblyopic children and remains integral in clinical practice (Repka et al., 2003; Buckle et al., 2019).

Our study further elucidates that short-term monocular deprivation of the amblyopic eye leads to a decrease in interocular suppression from the fellow eye toward the amblyopic eye (Lunghi et al., 2015a,b; Zhou et al., 2015; Chadnova et al., 2017). This shift in the excitation-inhibition balance in favor of the patched amblyopic eye aligns with previous research on alterations in neural interactions in the visual cortex(Lunghi et al., 2015a,b; Kim et al., 2017; Binda et al., 2018). Importantly, the decrease in neural response of the fellow eye after inverse occlusion challenges previous research demonstrating increased visual signals in the patched eye after monocular deprivation (Zhou et al., 2015, 2017). This inconsistency underscores the need for additional investigations to understand the distinct mechanisms underlying neural responses in the visual cortex following monocular deprivation of the fellow eye versus the amblyopic eye in amblyopic patients.

Our findings also highlight a correlation between amblyopic eye visual acuity and the change in interocular suppression after short-term inverse patching. This suggests that the severity of amblyopic eye visual acuity is intricately linked to neural plasticity (Buckle et al., 2019; Steinwurzel et al., 2020), proposing short-term inverse patching as a potential experimental alternative treatment for adolescent and adult patients with severe and refractory anisometropic amblyopia. However, the modest effects observed in psychophysical performance after short-term inverse patching necessitate further exploration, particularly in understanding the relationship between short-term and long-term reverse occlusion at both psychophysical and electrophysiological levels.

In conclusion, this study adds valuable insights into the intricate dynamics of neural responses to various patching interventions in amblyopic patients. The identification of age-dependent treatment efficacy and the correlation between visual acuity and neural plasticity emphasize the need for personalized approaches in amblyopia management. Future research endeavors should delve deeper into the connections between short-term and long-term inverse occlusion, shedding light on comprehensive treatment effects at both psychophysical and electrophysiological dimensions.

## Data availability statement

The original contributions presented in the study are included in the article/supplementary material, further inquiries can be directed to the corresponding authors.

## Ethics statement

The studies involving humans were approved by Zhongshan Ophthalmic Center Ethics Committee. The studies were conducted in accordance with the local legislation and institutional requirements. Written informed consent for participation in this study was provided by the participants’ legal guardians/next of kin.

## Author contributions

JH: Conceptualization, Data curation, Formal analysis, Investigation, Methodology, Validation, Visualization, Writing – original draft, Writing – review & editing. JC: Data curation, Formal analysis, Methodology, Software, Visualization, Writing – review & editing. YK: Conceptualization, Formal analysis, Funding acquisition, Methodology, Project administration, Supervision, Writing – review & editing. MY: Conceptualization, Funding acquisition, Project administration, Resources, Supervision, Writing – review & editing.

## References

[ref1] Abuleil D. McCulloch D. L. Thompson B. (2019). Older adults exhibit greater visual cortex inhibition and reduced visual cortex plasticity compared to younger adults. Front. Neurosci. 13:607. doi: 10.3389/fnins.2019.00607 31249506 PMC6582629

[ref2] Acar K. Kiorpes L. Movshon J. A. Smith M. A. (2019). Altered functional interactions between neurons in primary visual cortex of macaque monkeys with experimental amblyopia. J. Neurophysiol. 122, 2243–2258. doi: 10.1152/jn.00232.2019, PMID: 31553685 PMC6966320

[ref3] Baker D. H. Simard M. Saint-Amour D. Hess R. F. (2015). Steady-state contrast response functions provide a sensitive and objective index of amblyopic deficits. Invest. Ophthalmol. Vis. Sci. 56, 1208–1216. doi: 10.1167/iovs.14-15611, PMID: 25634977 PMC4334141

[ref4] Barnes G. R. Hess R. F. Dumoulin S. O. Achtman R. L. Pike G. B. (2001). The cortical deficit in humans with strabismic amblyopia. J. Physiol. 533, 281–297. doi: 10.1111/j.1469-7793.2001.0281b.x 11351035 PMC2278601

[ref5] Binda P. Kurzawski J. W. Lunghi C. Biagi L. Tosetti M. Morrone M. C. (2018). Response to short-term deprivation of the human adult visual cortex measured with 7T BOLD. eLife 7:e40014. doi: 10.7554/eLife.40014, PMID: 30475210 PMC6298775

[ref6] Buckle M. Billington C. Shah P. Ferris J. D. (2019). Treatment outcomes for amblyopia using PEDIG amblyopia protocols: a retrospective study of 877 cases. J. AAPOS 23, 98.e1–98.e4. doi: 10.1016/j.jaapos.2018.12.007, PMID: 30935990

[ref7] Chadnova E. Reynaud A. Clavagnier S. Hess R. F. (2017). Short-term monocular occlusion produces changes in ocular dominance by a reciprocal modulation of interocular inhibition. Sci. Rep. 7:41747. doi: 10.1038/srep41747, PMID: 28150723 PMC5288724

[ref8] Chen Z. Li J. Liu J. Cai X. Yuan J. Deng D.. (2016). Monocular perceptual learning of contrast detection facilitates binocular combination in adults with anisometropic amblyopia. Sci. Rep. 6:20187. doi: 10.1038/srep20187 26829898 PMC4735338

[ref9] Chen X. Wang Y. Nakanishi M. Gao X. Jung T. P. Gao S. (2015). High-speed spelling with a noninvasive brain-computer interface. Proc. Natl. Acad. Sci. U. S. A. 112, E6058–E6067. doi: 10.1073/pnas.1508080112, PMID: 26483479 PMC4640776

[ref10] Delorme A. Makeig S. (2004). EEGLAB: an open source toolbox for analysis of single-trial EEG dynamics including independent component analysis. J. Neurosci. Methods 134, 9–21. doi: 10.1016/j.jneumeth.2003.10.009, PMID: 15102499

[ref11] Ding Z. Li J. Spiegel D. P. Chen Z. Chan L. Luo G.. (2016). The effect of transcranial direct current stimulation on contrast sensitivity and visual evoked potential amplitude in adults with amblyopia. Sci. Rep. 6:19280. doi: 10.1038/srep19280 26763954 PMC4725886

[ref12] Epelbaum M. Milleret C. Buisseret P. Dufier J. L. (1993). The sensitive period for strabismic amblyopia in humans. Ophthalmology 100, 323–327. doi: 10.1016/S0161-6420(13)32170-8 8460000

[ref13] Fronius M. Cirina L. Ackermann H. Kohnen T. Diehl C. M. (2014). Efficiency of electronically monitored amblyopia treatment between 5 and 16 years of age: new insight into declining susceptibility of the visual system. Vis. Res. 103, 11–19. doi: 10.1016/j.visres.2014.07.018, PMID: 25130409

[ref14] Goodyear B. G. Nicolle D. A. Humphrey G. K. Menon R. S. (2000). BOLD fMRI response of early visual areas to perceived contrast in human amblyopia. J. Neurophysiol. 84, 1907–1913. doi: 10.1152/jn.2000.84.4.1907 11024083

[ref15] Gu L. Deng S. Feng L. Yuan J. Chen Z. Yan J.. (2020). Effects of monocular perceptual learning on binocular visual processing in adolescent and adult amblyopia. iScience 23:100875. doi: 10.1016/j.isci.2020.100875 32062454 PMC7021554

[ref16] Hensch T. K. (2005). Critical period plasticity in local cortical circuits. Nat. Rev. Neurosci. 6, 877–888. doi: 10.1038/nrn1787 16261181

[ref17] Hensch T. K. Quinlan E. M. (2018). Critical periods in amblyopia. Vis. Neurosci. 35:E014. doi: 10.1017/S0952523817000219, PMID: 29905116 PMC6047524

[ref19] Holmes J. M. Clarke M. P. (2006). Amblyopia. Lancet 367, 1343–1351. doi: 10.1016/S0140-6736(06)68581-4 16631913

[ref20] Holmes J. M. Lazar E. L. Melia B. M. Astle W. F. Dagi L. R. Donahue S. P.. (2011). Effect of age on response to amblyopia treatment in children. Arch. Ophthalmol. 129, 1451–1457. doi: 10.1001/archophthalmol.2011.179 21746970 PMC3217111

[ref21] Hu X. Qin Y. Ying X. Yuan J. Cui R. Ruan X.. (2021). Temporal characteristics of visual processing in amblyopia. Front. Neurosci. 15:673491. doi: 10.3389/fnins.2021.673491, PMID: 34149348 PMC8211088

[ref22] Hubel D. H. Wiesel T. N. (1968). Receptive fields and functional architecture of monkey striate cortex. J. Physiol. 195, 215–243. doi: 10.1113/jphysiol.1968.sp008455, PMID: 4966457 PMC1557912

[ref23] Hubel D. H. Wiesel T. N. (1970). The period of susceptibility to the physiological effects of unilateral eye closure in kittens. J. Physiol. 206, 419–436. doi: 10.1113/jphysiol.1970.sp009022, PMID: 5498493 PMC1348655

[ref24] Kelly K. R. Cheng-Patel C. S. Jost R. M. Wang Y. Z. Birch E. E. (2019). Fixation instability during binocular viewing in anisometropic and strabismic children. Exp. Eye Res. 183, 29–37. doi: 10.1016/j.exer.2018.07.013, PMID: 30006273 PMC7323568

[ref25] Kim H. W. Kim C. Y. Blake R. (2017). Monocular perceptual deprivation from interocular suppression temporarily imbalances ocular dominance. Curr. Biol. 27, 884–889. doi: 10.1016/j.cub.2017.01.063, PMID: 28262490

[ref27] Li J. Thompson B. Deng D. Chan L. Y. Yu M. Hess R. F. (2013). Dichoptic training enables the adult amblyopic brain to learn. Curr. Biol. 23, R308–R309. doi: 10.1016/j.cub.2013.01.059, PMID: 23618662

[ref28] Liu X. Y. Zhang J. Y. (2018). Dichoptic training in adults with amblyopia: additional stereoacuity gains over monocular training. Vis. Res. 152, 84–90. doi: 10.1016/j.visres.2017.07.002, PMID: 28736224

[ref29] Loudon S. E. Simonsz H. J. (2005). The history of the treatment of amblyopia. Strabismus 13, 93–106. doi: 10.1080/09273970590949818, PMID: 16020365

[ref30] Lunghi C. Berchicci M. Morrone M. C. Di Russo F. (2015a). Short-term monocular deprivation alters early components of visual evoked potentials. J. Physiol. 593, 4361–4372. doi: 10.1113/JP270950 26119530 PMC4594246

[ref31] Lunghi C. Burr D. C. Morrone C. (2011). Brief periods of monocular deprivation disrupt ocular balance in human adult visual cortex. Curr. Biol. 21, R538–R539. doi: 10.1016/j.cub.2011.06.004, PMID: 21783029

[ref32] Lunghi C. Emir U. E. Morrone M. C. Bridge H. (2015b). Short-term monocular deprivation alters GABA in the adult human visual cortex. Curr. Biol. 25, 1496–1501. doi: 10.1016/j.cub.2015.04.021, PMID: 26004760 PMC5040500

[ref33] Lunghi C. Sframeli A. T. Lepri A. Lepri M. Lisi D. Sale A.. (2018). A new counterintuitive training for adult amblyopia. Ann. Clin. Transl. Neurol. 6, 274–284. doi: 10.1002/acn3.698 30847360 PMC6389748

[ref34] Lygo F. A. Richard B. Wade A. R. Morland A. B. Baker D. H. (2021). Neural markers of suppression in impaired binocular vision. NeuroImage 230:117780. doi: 10.1016/j.neuroimage.2021.117780, PMID: 33503479 PMC8063178

[ref35] Malik S. R. Gupta A. K. Grover V. K. (1970). Occlusion therapy in amblyopia with eccentric fixation. Br. J. Ophthalmol. 54, 41–45. doi: 10.1136/bjo.54.1.41, PMID: 5417653 PMC1207582

[ref36] McKee S. P. Levi D. M. Movshon J. A. (2003). The pattern of visual deficits in amblyopia. J. Vis. 3, 380–405. doi: 10.1167/3.5.5, PMID: 12875634

[ref37] Min S. H. Mao Y. Chen S. He Z. Hess R. F. Zhou J. (2021). A clinically convenient test to measure binocular balance across spatial frequency in amblyopia. iScience 25:103652. doi: 10.1016/j.isci.2021.103652 35024586 PMC8733258

[ref38] Noordeng V. (1965). Occlusion therapy in amblyopia with eccentric fixation. Arch. Ophthalmol. 73, 776–781. 14302508 10.1001/archopht.1965.00970030778005

[ref39] Norcia A. M. Appelbaum L. G. Ales J. M. Cottereau B. R. Rossion B. (2015). The steady-state visual evoked potential in vision research: a review. J. Vis. 15:4. doi: 10.1167/15.6.4 PMC4581566 26024451

[ref40] Pediatric Eye Disease Investigator Group (2002). A randomized trial of atropine vs. patching for treatment of moderate amblyopia in children. Arch. Ophthalmol. 120, 268–278. doi: 10.1001/archopht.120.3.268 11879129

[ref41] Pelli D. G. (1997). The VideoToolbox software for visual psychophysics: transforming numbers into movies. Spat. Vis. 10, 437–442. 9176953

[ref42] Repka M. X. Beck R. W. Holmes J. M. Birch E. E. Chandler D. L. Cotter S. A. (2003). A randomized trial of patching regimens for treatment of moderate amblyopia in children. Arch. Ophthalmol. 121, 603–611. doi: 10.1001/archopht.121.5.603 12742836

[ref43] Rutstein R. P. Fuhr P. S. (1992). Efficacy and stability of amblyopia therapy. Optom Vis Sci 69, 747–754. doi: 10.1097/00006324-199210000-00001, PMID: 1436994

[ref44] Scheiman M. M. Hertle R. W. Beck R. W. Edwards A. R. Birch E. Cotter S. A.. (2005). Randomized trial of treatment of amblyopia in children aged 7 to 17 years. Arch. Ophthalmol. 123, 437–447. doi: 10.1001/archopht.123.4.437 15824215

[ref45] Schmidt K. E. Singer W. Galuske R. A. (2004). Processing deficits in primary visual cortex of amblyopic cats. J. Neurophysiol. 91, 1661–1671. doi: 10.1152/jn.00878.2003, PMID: 14668297

[ref47] Spiegel D. P. Baldwin A. S. Hess R. F. (2017). Ocular dominance plasticity: inhibitory interactions and contrast equivalence. Sci. Rep. 7:39913. doi: 10.1038/srep39913, PMID: 28071682 PMC5223198

[ref48] Steinwurzel C. Animali S. Cicchini G. M. Morrone M. C. Binda P. (2020). Using psychophysical performance to predict short-term ocular dominance plasticity in human adults. J. Vis. 20:6. doi: 10.1167/jov.20.7.6 PMC7424141 32634225

[ref49] Tang Y. Norcia A. M. (1995). An adaptive filter for steady-state evoked responses. Electroencephalogr. Clin. Neurophysiol. 96, 268–277. doi: 10.1016/0168-5597(94)00309-3, PMID: 7750452

[ref50] Tigges M. Boothe R. G. Tigges J. Wilson J. R. (1992). Competition between an aphakic and an occluded eye for territory in striate cortex of developing rhesus monkeys: cytochrome oxidase histochemistry in layer 4C. J. Comp. Neurol. 316, 173–186. doi: 10.1002/cne.903160204, PMID: 1315344

[ref51] Wiesel T. N. Hubel D. H. (1963). Single-cell responses in striate cortex of kittens deprived of vision in one eye. J. Neurophysiol. 26, 1003–1017. doi: 10.1152/jn.1963.26.6.1003, PMID: 14084161

[ref52] Zhou J. Baker D. H. Simard M. Saint-Amour D. Hess R. F. (2015). Short-term monocular patching boosts the patched eye’s response in visual cortex. Restor. Neurol. Neurosci. 33, 381–387. doi: 10.3233/RNN-140472, PMID: 26410580 PMC4923712

[ref53] Zhou J. Clavagnier S. Hess R. F. (2013). Short-term monocular deprivation strengthens the patched eye’s contribution to binocular combination. J. Vis. 13:12. doi: 10.1167/13.5.12, PMID: 23599416

[ref54] Zhou J. He Z. Wu Y. Chen Y. Chen X. Liang Y.. (2019). Inverse occlusion: a binocularly motivated treatment for amblyopia. Neural Plast. 2019, 1–12. doi: 10.1155/2019/5157628 PMC6444262 31015829

[ref55] Zhou J. Reynaud A. Kim Y. J. Mullen K. T. Hess R. F. (2017). Chromatic and achromatic monocular deprivation produce separable changes of eye dominance in adults. Proc. Biol. Sci. 284:20171669. doi: 10.1098/rspb.2017.1669 29142113 PMC5719170

